# Effect of Seed Priming on Early Development of Sorghum (*Sorghum bicolor* L. Moench) and *Striga hermonthica* (Del.) Benth

**DOI:** 10.1155/2014/134931

**Published:** 2014-07-23

**Authors:** Hussien M. Daffalla, Mohammed Mahgoub Hassan, Magdoleen G. Osman, Amani Hamad Eltayeb, Yassin Ibrahim Dagash, Migdam E. Abdel Gani

**Affiliations:** ^1^Commission for Biotechnology and Genetic Engineering, National Centre for Research, Khartoum, Sudan; ^2^Environment and Natural Resources Research Institute, National Centre for Research, Khartoum, Sudan; ^3^Sudan University of Science and Technology, College of Agricultural Studies, Khartoum, Sudan

## Abstract

*Striga hermonthica* is an obligate, root parasite, that limits cereal production in sub-Saharan Africa. Successful control depends on eliminating its seed reserves in soil, thereby preventing parasitism. Two experiments were conducted to evaluate the effects of salinity on germination traits and seedling growth of sorghum (cultivar Wad Ahmed) and *S. hermonthica*. The experiments were conducted in a factorial arrangement on the basis of completely randomized design (CRD) with 4 replications. In the first experiment, sorghum height, leaf area, and shoot and root dry weights were examined. The results displayed that, with increasing salinity level, leaf area and dry biomass were increased, while the height was decreased. In the second experiment, *Striga* germination and haustorium initiation percentages were examined. Among all salts, C_2_H_4_O_2_·NH_3_ inhibited *Striga* germination (0–15%) during conditioning or (0–25%) at germination compared to the control (75%). However, salt MgSO_4_·7H_2_O improved germination during conditioning up to 70%, while during germination CH_3_COONa·3H_2_O recorded 65% germination. Regarding haustoria initiation, results showed that C_2_H_4_O_2_·NH_3_ at all concentrations inhibits haustorium formation by 100%, while CH_3_COONa·3H_2_O at 10 µM improved haustorium formation up to 64% but still below the control (70%). Osmotic potential may significantly affect germination and radicle elongation of the parasitic weed.

## 1. Introduction

Sorghum (*Sorghum bicolor* (L.) Moench; Poaceae) was domesticated in different areas of Africa. It is the fifth most important cereal crop in the world after wheat, rice, corn, and barley. Sorghum possesses a variety of anatomical, morphological, and physiological features that enable it to survive in water-limited environments [1]. Salinity is the most important environmental factor affecting the crop production in many parts of the world. Salinity has reached a level of 19.5% of all irrigated land and 2.1% of dry-land agriculture worldwide [2]. It reduces the ability of plants to utilize water and causes a reduction in growth rate, as well as changes in plant metabolic processes. One of the reasons of salinity is the high concentration of cations such as sodium, calcium, and magnesium whereas chloride, phosphate, and nitrate as anions. The effect of salinity on plant growth is a complex trait that involves osmotic stress, ion toxicity, mineral deficiencies, and physiological and biochemical perturbations [3]. The plants that grow in saline soils have diverse ionic compositions and a range in concentrations of dissolved salts. Salt in soils affects seed germination as influenced by the total concentration of dissolved salt as well as by the type of salt involved. Netondo et al. [4] reported that the increase of NaCl concentration significantly reduced the relative shoot growth rate and shoot dry weight of sorghum. Leaf water potential, osmotic potential, leaf pressure potential, and relative water content also declined significantly with the increase of salt stress. The overall effect of salinity on plants is the eventual shrinkage of leaf size, which leads to death of the leaf and finally the plant. Salinity may also cause reduced ATP and growth regulators in plants. Soil salinity is known to suppress the growth of most crop species, but considerable differences in salinity tolerance exist between species [5]. Presoaking or priming seeds of a number of crops has improved germination, seedling establishment, and, in some cases, stimulated vegetative growth and hence crop yield [6]. Research has shown that salt priming may be a reliable method for making crops more resilient in saline growing conditions. Priming is simply exposing the unplanted seeds of a plant to a solution of salt water and allowing the salt content to soak into the seed. Theoretically, this early exposure to saline conditions will allow the seed to adapt to such conditions and thus tolerate high salinity concentrations in soil as a full-grown plant.

*Striga*, an obligate root parasite, has become the greatest biological constraint on food production in Africa.* Striga* would be dependent on the host for supplies of water, mineral salts, and sugar as minimal requirement of the parasite in order to develop a shoot system and achieve normal growth [7]. Crop yield loss can reach levels of 100% due to heavy* Striga* infestation. In Sudan,* S. hermonthica* is a common weed in most of cereals' cultivated areas throughout the country. Many potential control methods were developed against the parasite including physical, cultural, chemical, and biological. However, so far these methods have only a limited impact on controlling* Striga* and today there is no single control method that can effectively solve this problem. Most of these methods are either time consuming or unaffordable by small subsistent farmers. The objectives of the present study were (i) to evaluate the effects of different salts on early seedling growth of sorghum and (ii) to determine the effect of different salts on early developmental stages of* Striga hermonthica*.

## 2. Materials and Methods

Series of laboratory and green house experiments were undertaken to investigate the effects of seed priming and salinity on germination growth of sorghum and haustorium initiation of* Striga hermonthica*. The experiments were conducted in 2013 at the Environment and Natural Recourses Research Institute (ENRRI), Khartoum, Sudan.

### 2.1. Experimental Materials

#### 2.1.1. Plant Materials

This experiment was done as a factorial experiment in the base of completely randomized design with four replications. Seeds of sorghum cultivar Wad Ahmed were obtained from the Arab Sudanese Seeds Company limited, Khartoum, Sudan.* S. hermonthica* seeds were collected in 2004 from infected sorghum fields at the Gezira Research Station Farm, Sudan.

#### 2.1.2. Test Solutions

Three salt types, namely, magnesium sulphate (MgSO_4_·7H_2_O), ammonium acetate (C_2_H_4_O_2_·NH_3_), and sodium acetate trihydrate (CH_3_COONa·3H_2_O), were utilized in this study and abbreviated as Mg, NH, and Na, respectively. The salts were prepared in four concentrations (0 as a control, 60, 80, and 100 *μ*M) for the experiment of sorghum. For* Striga* experiment lower concentrations of the same salts were prepared (10, 20, 30, 40, and 50 *μ*M) in addition to 0 *μ*M as control.

A synthetic strigolactone analog (GR24) was provided by Professor B. Zwanenberg, the University of Nimijhen, the Netherlands. A stock solution of the stimulant was prepared by dissolving 1 mg in 1 mL of acetone and completing to volume (100 mL) with sterile distilled water. The solution was kept in a fridge at 5°C till used.

The 2, 6-dimethoxy-p-benzoquinone (DMBQ) was a gift from Dr. Sugimoto, Y. from Kobe University, Japan. A stock solution (100 *μ*M) was prepared by dissolving 1.68 mg in 1 mL of acetone and completing to volume (100 mL) with sterile distilled water.

### 2.2. Methodology

#### 2.2.1. Laboratory Experiments

*Surface Disinfection, Conditioning, and Germination of Striga Seeds. Striga* seeds were surface sterilized as described by Babiker [7]. Briefly, the seeds were soaked in 70% ethanol for 2 min and rinsed three times with distilled water. Subsequently the seeds were immersed in 1% NaOCl solution for 3 min with continuous agitation and then thoroughly washed with sterilized distilled water. Floating seeds were discarded. The remaining seeds were air dried under laminar flow hood and then kept in sterilized vials at ambient temperature till used. Disinfected seeds were conditioned as described by Babiker [7]. Glass-fiber filter papers (GF/C) discs (8 mm diameter) were cut, wetted thoroughly with water, and placed in an oven at 100°C for 1 h to be sterilized and ready for further use. The glass-fiber disks were placed on moistened glass filter paper in Petri dishes (9 cm). About 25–30 sterilized* Striga* seeds were gently sprinkled on each glass-fiber disc. The dishes were sealed with parafilm, covered with black polythene bags, and incubated at 30°C in the darkness for 10 days. Then* Striga* seeds were treated with GR24 at 0.1 mg/L and reincubated under the same conditions for 24 h.

*Effects of Different Salts Applied at Termination on S. hermonthica Seeds Germination in Response to GR24.* The sterilized discs, placed in 9 cm Petri dishes lined with glass fiber filter papers (GF/C), were moistened with 5 mL distilled water. About 25–30 surface disinfected* Striga* seeds were sprinkled on each of the glass fiber discs in each petri dish. The dishes, sealed with parafilm, were placed in black polythene bags and incubated at 30°C in the dark for 10 days. Then* Striga* seeds were treated with mixture of different levels of salts and 0.1 mg/L GR24 and reincubated and germination rate was determined after 24 h.* Striga* seeds conditioned in water and treated with GR24 were included as a control.

*Effect of Different Salts, Applied During Conditioning on Striga Seeds Germination in Response to GR24.* Three salts and their concentrations were evaluated for their ability to inhibit GR24-induced germination of* S. hermonthica* seeds.* Striga* seeds were conditioned in salts as shown above. The discs containing* Striga* seeds were treated with 20 *μ*L of GR24 at 0.1 mg/L or distilled water. The seeds were reincubated and examined for germination as described above.* Striga* seeds conditioned in distilled water and similarly treated with GR24 were included as control for comparison.

*Effects of Different Salts on Haustorial Initiation in Striga. Striga* seeds, placed on 8 mm glass fiber discs, conditioned in the presence of each concentration of each salt, were dapped on filter papers and transferred to sterile Petri dishes (as mentioned above). The discs containing* Striga* seeds were treated, each, with 20 *μ*L GR24 (0.1 mg/L) to induce germination. The Petri dishes, sealed with parafilm and placed in black polythene bags, were incubated in the dark at 30°C for 48 h. The discs containing the germinated* Striga* seeds dapped on a filter paper were placed and inverted top-down on similar discs without* Striga* seeds. The Pairs of discs were treated with 40 *μ*L solution of DMBQ (100 *μ*M). A germinated* Striga* seed (germiling) resulting from seeds conditioned in distilled water and similarly treated with DMBQ was included as control for comparison. The Petri dishes, sealed with parafilm and placed in polyethylene bags, were incubated in the dark at 30°C for an additional 24 h and then examined for haustorium initiation using a binocular stereomicroscope.

### 2.3. Pot Experiment

*Seed Priming, Plant Cultivation, and Salt Stress Induction on Sorghum.* Sorghum cv. Wad Ahmed seeds were surface sterilized with 0.5% of sodium hypochlorite for 5 min and then thoroughly washed with tap water. To assess the priming effects on sorghum growth, seeds were presoaked in 50 mL of each salt solution in 60, 80, and 100 *μ*M for 12 h. Nonprimed seeds prepared by immersing seeds in distilled water were included as control. The seeds were sown in plastic pots (12 cm diameter and 22 cm height) filled with 7 kg mixture of a river silt: sand (2 : 1, v/v) soil with drainage holes at the bottom to avoid water logging. Before sowing the seeds (8/pot), 15 mL of each concentration of each salt was applied to each pot. In the control treatment, distilled water was used. Seeds were sown in pots and watered to field capacity (1500 mL) with deionized water for up to 56 days. The field capacity was determined on a soil weight basis (%21.34, w/w). Pots were irrigated daily to the desired level to maintain soil moisture levels to field capacity.

### 2.4. Analysis of Morphological Parameters

Morphological parameters, including plant height (cm, from the base to the tip of the longest leaf), leaf numbers, and leaf area, were measured at 3 and 8 weeks after sowing (WAS). The leaf areas for each seedling were calculated as maximum width × maximum length × 0.75, where the term in this formula approximates the oval shape of the leaves [8]. Dry biomass of shoot and root was obtained by the end of experiment (8 WAS). Plants were harvested and each was separated into shoot and root systems (at the pot surface). Root systems were washed carefully to insure no roots were lost and then placed on absorbent paper. The separated plant materials were oven-dried at 80°C for 48 h until a constant dry weight is obtained, and the dry weights (DW) were recorded. Percentage of relative dry weight (RDW%) of tissues was also obtained by comparing salinized plants biomass to those of control plants as salinized tissue dry weight/control dry weight × 100 L [9]. Root: plumule ratio was calculated as dry weight for roots/dry weight for top of plant.

### 2.5. Statistical Analysis

In all experiments, treatments were arranged in a randomized complete design with 4 replicates. Data on percentage of germination, growth, and haustorium initiation were calculated for each treatment. All data were subjected to two-way or three-way analysis of variance (ANOVA). Mean separation between treatments was determined using LSD test at the 0.05 level [10].

## 3. Results and Discussion

Plant growth is an irreversible increase in mass that is typically associated with an increase in volume. Modifications in behavior of species under salinization are determined by analysis of growth parameters under controlled environment. So, in this study, volume (length and size) along with mass (dry biomass) parameters of sorghum seedlings was analyzed under salinity condition. The results of treated sorghum seedlings with different salts indicated insignificant effects either negatively on shoot length or positively on leaf area and dry biomass.

### 3.1. Soot Length and Leaf Area Analysis

**Figure 1:**
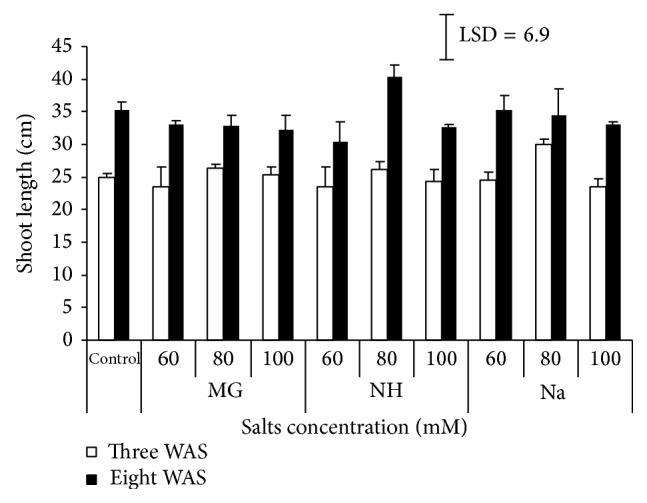
Effect of different salts levels on sorghum shoot length 3 WAS (white bar) and 8 WAS (black bar). WAS: weeks after sowing, MG: MgSO_4_·7H_2_O, NH: C_2_H_4_O_2_·NH_3_, and Na: CH_3_COONa·3H_2_O. Vertical bar represents standard error, LSD value (*P* ≤ 0.05) for comparison between treatments.

The results indicated varying degree of inhibitory effect of salts on shoot growth (Figure 1). At 3 WAS and 8 WAS, sorghum seedling height was improved through time, irrespective to salt types. At 8 WAS, plants reach the maximum length recorded (40.4 cm) at 80 *μ*M NH compared to height in control (35.3 cm).

The effect of salinity on sorghum growth could be wrapped up; as salinity increased, seedling length slightly decreased. These findings are in agreement with other works [11–13] reported on sorghum. Seedling growth encompasses the division and expansion of cells. Excess of salt in growth medium restricts the availability of water to plant. This restriction results in dehydration of cytoplasm which in turn affects the metabolism of the cells and ultimately reduces the growth of plant.

**Figure 2:**
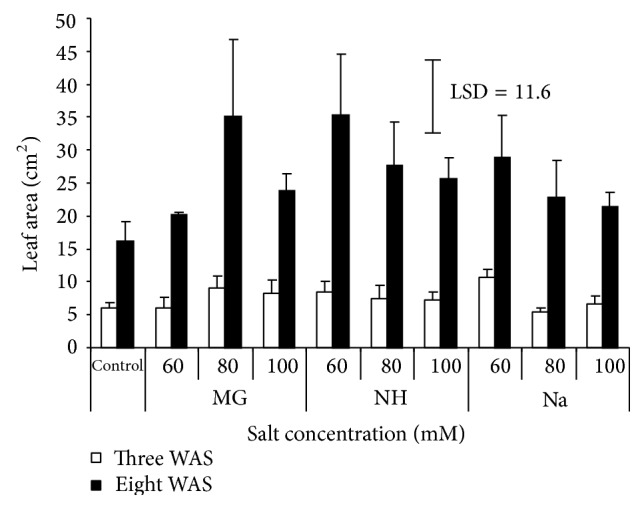
Effect of different salts levels on sorghum leaf area after 3 WAS (white bar) and 8 WAS (black bar). WAS: weeks after sowing, MG: MgSO_4_·7H_2_O, NH: C_2_H_4_O_2_·NH_3_, and Na: CH_3_COONa·3H_2_O. Vertical bar represents standard error, LSD value (*P* ≤ 0.05) for comparison between treatments.

**Figure 3:**
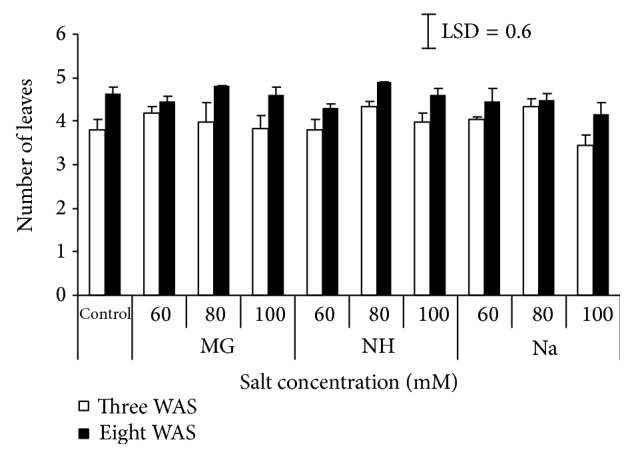
Effect of different salts levels on the number of leaves after 3 WAS (white column) and 8 WAS (black column). WAS: weeks after sowing, MG: MgSO_4_·7H_2_O, NH: C_2_H_4_O_2_·NH_3_, and Na: CH_3_COONa·3H_2_O. Vertical bar represents standard error, LSD value (*P* ≤ 0.05) for comparison between treatments.

Salinity is reported to affect the number, form, size, and growth rate of leaves. In the present study, the presence of salt enlarged the individual leaf area compared to nonsalinized plants (Figure 2). Generally, leaf area increased gradually from first to last measuring. Final reading at 8 WAS showed that the rise in Mg concentration to 100 *μ*M increased the area per leaf, while with 80 *μ*M of both NH and Na, it represents the optimum level for the broad leaf area. The maximum leaf area recorded was 35.3 cm^2^ by seedlings treated with 80 *μ*M NH compared to control (16.2 cm^2^) which was the minimum value obtained. In contrary to this study, Bashir et al. [11] and Jafari et al. [14] reported that salinity decreased leaf area of sorghum. However, Mane et al. [15] observed increase in leaf area per plant of the grass* Pennisetum alopecuroides* treated with NaCl. The increase in leaf area of the grass under the influence of salinity might be due to the production of toxic substances mainly involving reactive oxygen species. On the other hand, the number of leaves slightly increased when comparing the first counting (3 WAS) with the last one (8 WAS). In all salt types, the middle concentration (80 *μ*M) represented the optimum concentration (Figure 3). Moreover, the number of leaves of sorghum was reported to be significantly affected by NaCl application [14]. Sadeghi and Shourijeh [8] measured the number of leaves in 2 different times and found that the number of leaves was decreased from first to last counting.

### 3.2. Dry Weight Analysis

**Figure 4:**
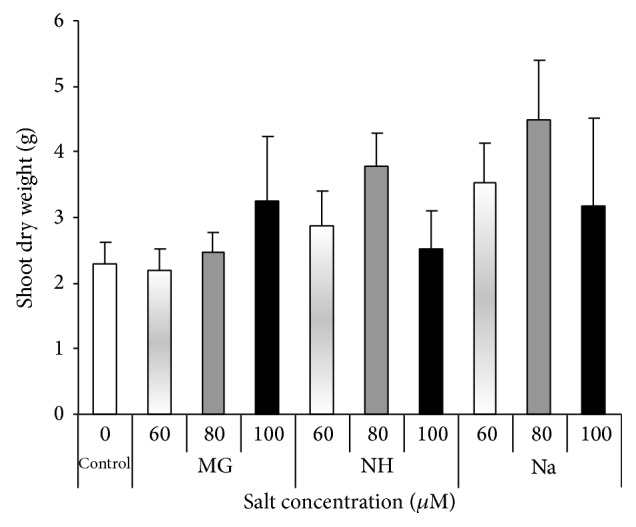
Effects of different salts levels on sorghum shoot dry weight after 8 WAS. WAS: weeks after sowing, MG: MgSO_4_·7H_2_O, NH: C_2_H_4_O_2_·NH_3_, and Na: CH_3_COONa·3H_2_O. Vertical bar represents standard error; statistical differences were not detected by analysis of variance.

**Figure 5:**
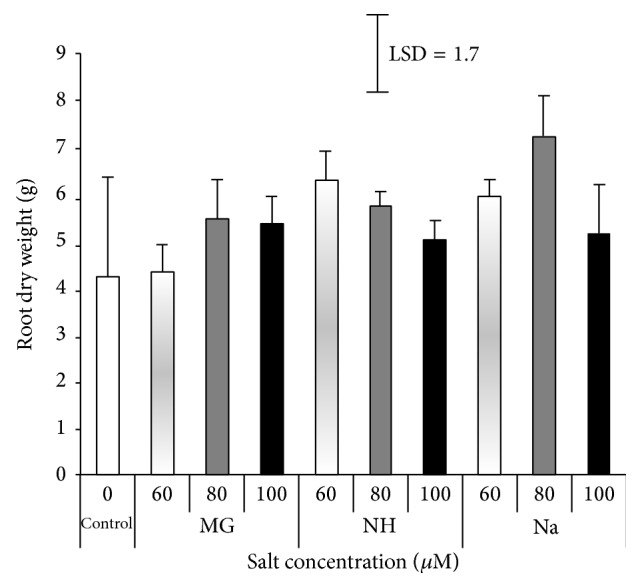
Effects of different salts levels on sorghum root dry weight after 8 WAS. WAS: weeks after sowing, MG: MgSO_4_·7H_2_O, NH: C_2_H_4_O_2_·NH_3_, and Na: CH_3_COONa·3H_2_O. Vertical bar represents standard error; LSD value (*P* ≤ 0.05) for comparison between treatments.

The presence of different salts enhanced the root and shoot dry biomass. Compared to the control, shoot and root dry matter increased at all concentrations (Figures 4 and 5). The highest shoot dry weight was 4.5 g with 80 *μ*M Na while the lowest value was 2.2 g with 60 *μ*M Mg compared to the control (2.3 g). The highest root dry weight was 7.4 g with 80 *μ*M Na while the lowest value was 4.3 g on the control. The optimal concentration for dry biomass increment varies between shoot and root and between salt types. The concentration 80 *μ*M of NH or Na and Mg or Na was optimal for shoot and root dry biomass, respectively, while 100 *μ*M of Mg and 60 *μ*M of NH were optimal for shoot and root biomass, respectively (Figures 4 and 5).

**Figure 6:**
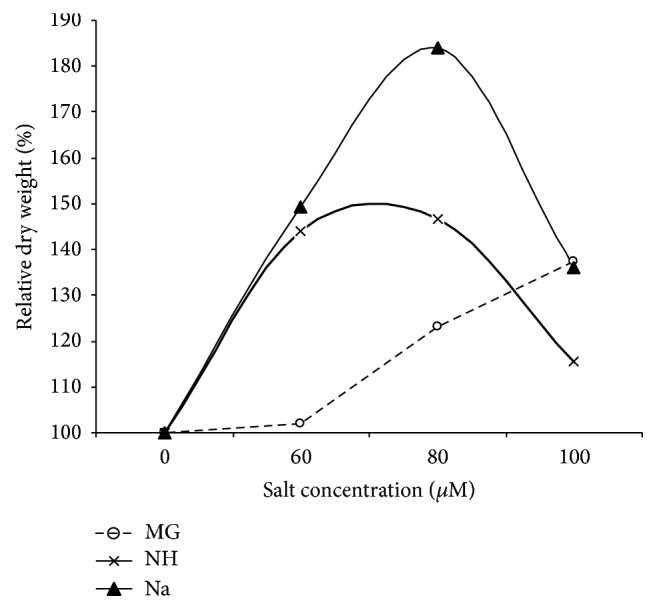
Effect of different salts levels on relative dry weight (%) of sorghum seedling after 8 WAS. WAS: weeks after sowing, MG: MgSO_4_·7H_2_O, NH: C_2_H_4_O_2_·NH_3_, and Na: CH_3_COONa·3H_2_O.

The relative dry weight (RDW%) of sorghum seedling was calculated by the end of the experiment (Figure 6). The results indicated a gradual increment in RDW with increasing salinity concentration of NH and Na up to 80 *μ*M. However, with Mg, the RDW continued to increase with the increase of salinity concentration to 100 *μ*M.

**Figure 7:**
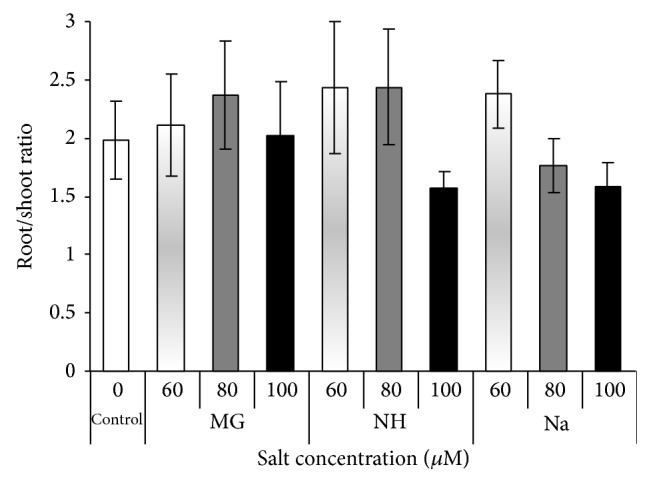
Effect of different salts levels on sorghum root/shoot ratio after 8 WAS. WAS: weeks after sowing, MG: MgSO_4_·7H_2_O, NH: C_2_H_4_O_2_·NH_3_, and Na: CH_3_COONa·3H_2_O. Vertical bar represents standard error; statistical differences were not detected by analysis of variance.

The root/shoot ratio of plants under control condition is considered as a “normal” ratio, to which saline plants are compared. Any changes from this normal level would be an indication of a change in the overall health of plant. Salinized seedling with root: shoot higher than control considered healthy while seedlings with ratio under normal level regard as sensitive to salt type or level. According to this, seedlings treated with Mg (all levels), NH (60, 80 *μ*M), and Na (60 *μ*M) recorded a good root/shoot ratio with improved growth under salinity (Figure 7). Seedlings treated with NH (100 *μ*M) and Na (80, 100 *μ*M) are considered as negatively affected by salinity compared to control.

The general idea from the results on sorghum biomass dry weight is in agreement with previous work reported by Kader and Jutzi [16] as salt increased dry biomass. This is in contrast with other reports which highlighted that shoot dry weight reduced as a result of salinity treatment [13, 17] or had no effect [12, 18].

Plant biomass production depends on the accumulation of carbon products in photosynthesis. Therefore, when the size of leaf surface increased the rate of photosynthesis per leaf area increased consequently [8]. In this study, salinity enhanced dry biomass due to an increase in photosynthesis as compared to increases in leaf expansion. This is in agreement with previous research findings in sorghum [12].

Investigation salinity tolerance of a crop during early seedling growth is critical for the establishment of plants under arid saline soil regions [16]. Tolerance to salt of many agronomic crops has been determined including sorghum. Sorghum was classified as moderately salt tolerant plant [9, 17]. However, same plant species respond unpredictably to salinity. Some studies reported variation in salinity tolerance between sorghum cultivars [12, 18].

It is apparent that priming of seeds of different crops can alleviate the adverse effect of salinity stress on germination and seedling establishment and, in some cases, it can enhance crop yield [6]. The presoaking of seeds allows the hydration of membranes and proteins and the initiation of various metabolic systems. These are arrested when the seeds are dried or moisture is withheld but recommence when the seeds imbibe water for the second time [19]. The presowing treatments cause initiation of the early metabolic processes and the redrying of seeds arrests, but does not reverse, the initial stages of germination so that on the availability of suitable conditions, the time taken to germinate is reduced [19]. Conrath et al. [20] reported that the primed plants display a faster and stronger activation of the various defense responses that are induced following attack by pathogens, insects, and various abiotic stresses.

### 3.3. Effects of Salts Applied during Conditioning on* S. hermonthica* Seeds Germination

**Figure 8:**
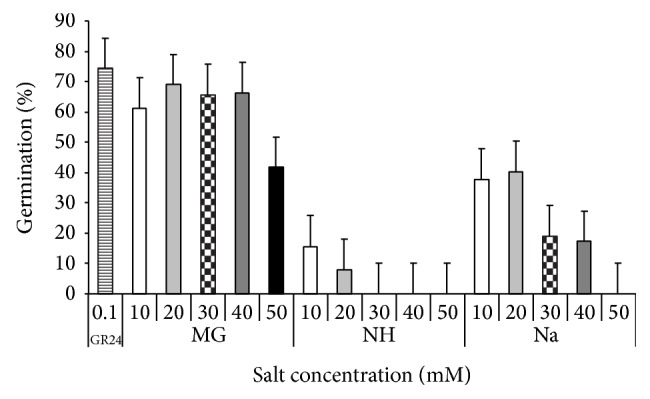
Effect of different salts concentrations applied during conditioning on* S. hermonthica* seeds germination in response to GR24 (strigolactone). MG: MgSO_4_·7H_2_O, NH: C_2_H_4_O_2_·NH_3_, and Na: CH_3_COONa·3H_2_O. Vertical bar represents standard error.

The results indicated that there was considerable variation in germination response of* Striga* to salt treatments (Figure 8). GR24 applied to seeds conditioned in water sustained the highest germination (73%). All salt treatments, applied during conditioning, reduced germination significantly, except Mg salt, irrespective to their concentrations. They reduced germination by 45–100% as compared to the control. Furthermore, NH salt was more suppressive to* S. hermonthica* germination than Na. However,* Striga* seeds conditioned in presence of Mg sustained the highest germination as compared to other salts, irrespective to their concentration.

### 3.4. Effects of Salts on GR 24-Induced Germination of* Striga*

**Figure 9:**
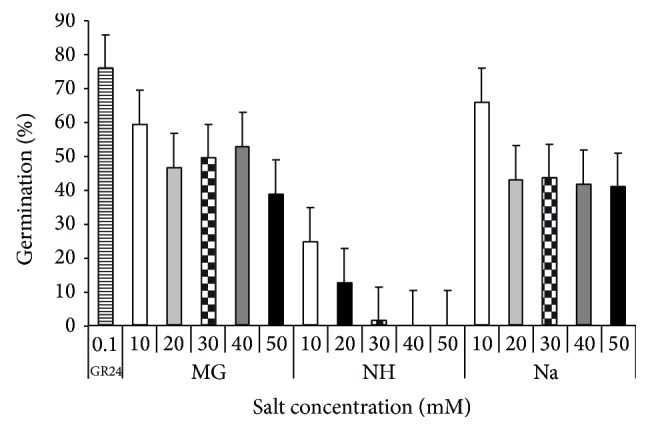
Effect of different salts concentrations, applied at germination on* S. hermonthica* seeds germination in response to GR24 (strigolactone). MG: MgSO_4_·7H_2_O, NH: C_2_H_4_O_2_·NH_3_, and Na: CH_3_COONa·3H_2_O. Vertical bar represented standard error.

*S. hermonthica* seeds, previously conditioned in presence of salts, showed variable response to GR24. Results revealed that* Striga* seeds treated with distilled water displayed negligible germination in all experiments (Figure 9). GR24 applied to seeds conditioned in water induced the highest germination (75%). All concentrations of each salt decreased* S. hermonthica* germination in response to GR24 in comparison with the corresponding aqueous controls. Among all salts studied, NH was the most inhibitor to* Striga* seed, irrespective to their concentration. The highest concentrations of the salt (40 and 50 *μ*M) completely inhibited germination, while the lowest salt concentrations (10–30 *μ*M) decreased germination by 73–96% compared to the corresponding control. Mg salt sustained the highest germination in response to GR24 as compared to other salts or control.

### 3.5. Effects of Salinity on Haustorial Initiation in* S. hermonthica*

**Figure 10:**
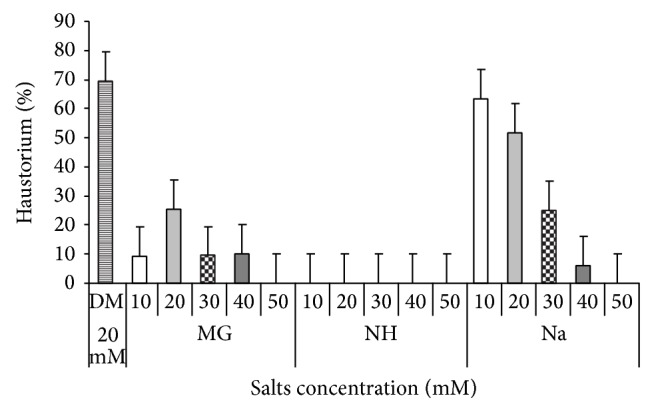
Effect of different salts levels on* Striga* haustorium initiation in response to DM (DMBQ: 2, 6-dimethoxy-p-benzoquinone). MG: MgSO_4_·7H_2_O, NH: C_2_H_4_O_2_·NH_3_, and Na: CH_3_COONa·3H_2_O. Vertical bar represented standard error.

DMBQ applied to* Striga* germilings resulted from seeds previously conditioned in water and GR24 induced 70% haustoria (Figure 10). All salts reduced haustoria significantly as compared to the control. The results showed that NH absolutely inhibited haustoria initiation in response to haustorium factor, while Mg caused a significant reduction in haustorial initiation compared to the control. Na at the lowest concentrations, on the other hand, promoted haustorium induction albeit not significantly, compared to the control. However, Na at the lowest concentrations (10 and 20 *μ*M) showed the least effect on* Striga* haustorium in response to DMBQ. They reduced haustorium by 14–29% compared to the control.

Several factors influence germination of* Striga* in the soil including temperature, moisture, pH, nutrients, soil type, and stimulants produced by host plants. A negative relationship was observed between salt levels and germination percentage of* Striga* seeds during or after conditioning and haustorium. Hassan et al. [21] reported that* Striga* and* Orobanche* spp. seeds rarely germinated when incubated in NaCl solution. That soil saturated with 75 *μ*M NaCl resulted in complete absence of* Striga* emergence. While sorghum treated with 50 *μ*M NaCl sustained the least* Striga* infestation, it reduced* Striga* infestation by 74 and 55% after 45 and 60 days, respectively. The effect of salinity on seed germination could be due to the toxic effect of salts on seeds or to the osmotic effect that prevents the seeds from imbibition [22]. Therefore, the effect of salinity on germination of* Striga* seeds may be due to some biochemical changes occurring within the seeds. Such biochemical changes lead to decreased seed germination and were postulated upon as a specific ion toxicity of the salts rather than osmotic potential on the seeds.

Haustorium initiation in response to DMBQ was inhibited by the higher level of salt. Moreover, the inhibitory effects showed dependence on the level of salt used and the source of the haustorium factor. Inhibition of haustorium initiation in* Striga* by salt may be attributed to phytotoxic substances, inhibitors, or extracellular enzymes that degrade and/or reduce production of H_2_O_2_ in* Striga* radicle tip [23]. Salinity exerts its undesirable effects through osmotic inhibition and ionic toxicity [24].

The mechanisms for seed priming that trigger the changes in the processes of germination and seedling growth are not fully understood. So, effect of salts priming on salt tolerance of sorghum seeds still requires more investigations at biochemical level before applying the method at field. Moreover, studying the effect of salts priming on sorghum root exudates is prerequisite since haustoria development of* Striga* depends on these solutes. It could be concluded that there is an optimum level of NH_4_C_2_H_3_O_2_ salt (mostly 80 *μ*M) producing the best morphological growth parameters of sorghum. Increasing the concentration beyond that level is associated with decreases in all studied characters. At the same time, application of NH_4_C_2_H_3_O_2_ salt to* Striga* inhibited seeds germination and haustorium formation which was the main issue in controlling witchweed infection.
